# Modeling nuclear energy’s future role in decarbonized energy systems

**DOI:** 10.1016/j.isci.2023.105952

**Published:** 2023-01-09

**Authors:** John Bistline, Shannon Bragg-Sitton, Wesley Cole, Brent Dixon, Erich Eschmann, Jonathan Ho, Augustine Kwon, Laura Martin, Caitlin Murphy, Christopher Namovicz, Andrew Sowder

**Affiliations:** 1 Electric Power Research Institute, 3420 Hillview Avenue, Palo Alto, CA 94304, USA; 2 Idaho National Laboratory, 1955 N Fremont Avenue, Idaho Falls, ID 83415, USA; 3 National Renewable Energy Laboratory, 15013 Denver W Pkwy, Golden, CO 80401, USA; 4 U.S. Environmental Protection Agency, 1200 Pennsylvania Avenue NW, Washington, DC 20004, USA; 5 U.S. Energy Information Administration, 1000 Independence Avenue SW, Washington, DC 20585, USA

**Keywords:** Energy management, Energy Modeling, Energy systems, Engineering

## Abstract

Increased attention has been focused on the potential role of nuclear energy in future electricity markets and energy systems as stakeholders target rapid and deep decarbonization and reductions in fossil fuel use. This paper examines models of electric sector planning and broader energy systems optimization to understand the prospective roles of nuclear energy and other technologies. In this perspective, we survey modeling challenges in this environment, illustrate opportunities to propagate best practices, and highlight insights from the deep decarbonization literature on the range of visions for nuclear energy’s role. Nuclear energy deployment is highest with combinations of stringent emissions policies, nuclear cost reductions, and constraints on the deployment of other technologies, which underscores model dimensions related to these areas. New modeling capabilities are needed to adequately address emerging issues, including representing characteristics and applications of nuclear energy in systems models, and to ensure the relevance of models for policy and planning as deeper decarbonization is explored.

## Graphical abstract

**Figure:**
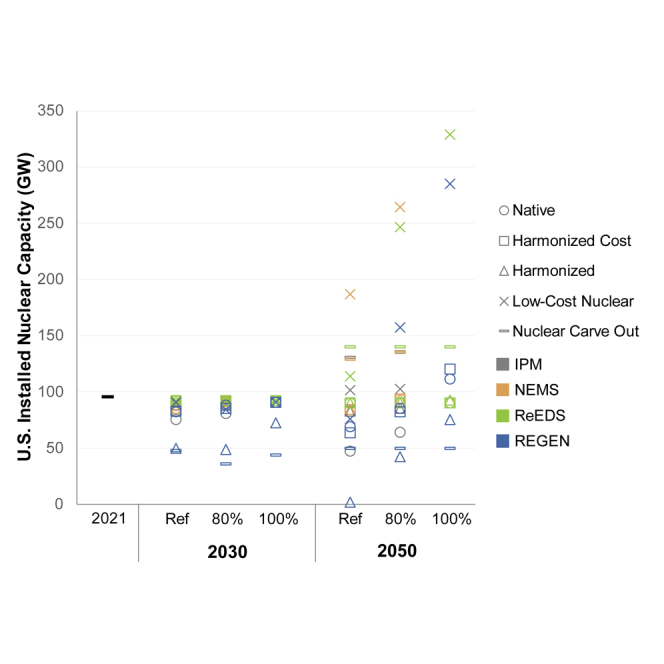

## Introduction

**Figure 1:**
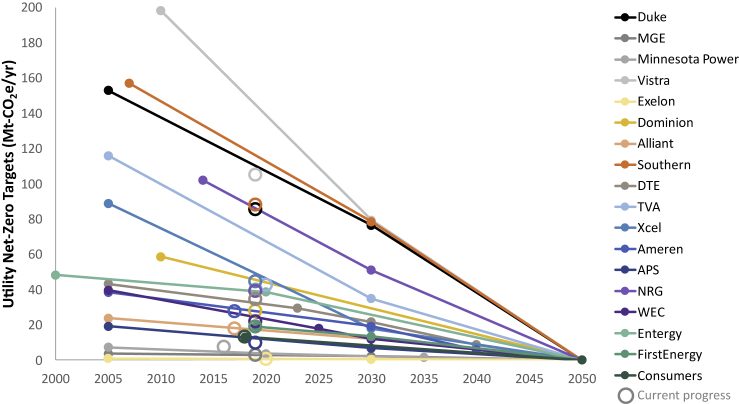
Electric sector targets and historical CO_2_ emissions in the US for companies with net-zero goals Data come from company filings and a database of electric sector decarbonization targets.^4^ These companies together represent about 40% of current US power sector CO_2_ emissions.

In response to the risks posed by climate change and air pollution,^1^^,^^2^ many governments and companies are making decarbonization pledges, including electric sector commitments to net-zero emissions (Figure 1). As a carbon-free energy resource, nuclear energy can play a role in meeting decarbonization goals, but there is uncertainty about the size of these contributions. This uncertainty is because of questions concerning nuclear energy’s competitiveness, the availability of new nuclear technologies, project execution risk, and unknowns about the stringency, form, and timing of economy-wide emission reductions policies, which can lead to growth in demand from electrification and non-power applications of nuclear energy.^3^

At the same time, updated global forecasts in 2022 exhibit declines in natural gas use and increases in renewables and nuclear relative to 2021 projections,^5^ including announcements of new nuclear projects in Japan, France, China, and US and delayed retirements in others. There are several drivers contributing to these trends with regional variation. Some countries are placing emphasis on energy security, especially for regions with greater dependence on imported fossil fuels, in wake of the Russian invasion of Ukraine.^6^ Natural gas reductions in Europe are related to supply constraints and in the US (and other countries with greater domestic supplies) are related to rising prices and new policies. Increases in nuclear are driven by decarbonization in some places, by high and/or volatile fossil fuel prices, and by the war in Europe.

To understand these dynamics, long-term electric sector capacity planning and broader energy systems optimization models are used for informing planning, technology assessment, and policy analysis with a range of different model structures, input assumptions, and uses. Many models were built decades ago, but the current market and projected futures look different from the environments in which these models were built—deeper decarbonization, higher shares of variable renewable energy, and greater cross-sector energy systems integration. Adequately characterizing technological capabilities and policy conditions are important for meeting objectives of decision-makers for affordable, reliable, sustainable, resilient, secure, and equitable energy systems.

Earlier literature examines broader needs for appropriately representing economic characteristics and technical details of energy system resources,^7^ including papers focusing on variable renewable energy,^8^ energy storage,^9^ and hybrid resources.^10^ There are also papers looking at scenario analyses focusing on nuclear energy.^11^^,^^12^^,^^13^ This paper is the first to provide an assessment of model needs related to nuclear energy for long-term electric sector capacity planning and broader energy systems optimization models.

This article examines modeling challenges related to nuclear energy and illustrates opportunities to propagate best practices. The objective is to bridge the interpretability gap between modelers and consumers of model outputs: Such tools should yield insights that are explainable to and trusted by model users including policymakers, industry, technology developers, and the public and firmly grounded in transparent and rigorous analysis approaches. We focus on existing and new nuclear for specificity, but many points broadly apply to the use of models to generate insights under deep decarbonization.

## Considerations for modeling nuclear energy

Many recent factors have focused attention on nuclear energy’s prospective future role. First, deep decarbonization policy and company targets in the power sector and across the economy have increased interest in low-, zero-, or negative-emissions technologies such as variable renewable energy, energy storage, carbon-capture-equipped capacity, nuclear energy, and others.^14^ Incentives for accelerating decarbonization have increased globally in 2022, including the Inflation Reduction Act in the US and REPowerEU Plan in Europe. Second, the aging fleet of coal and nuclear capacity in many countries raise questions about timelines for retirement and replacement capacity.^15^ Third, progress in advanced nuclear reactor designs and plans for deployment have heightened expectations for nuclear energy’s role, including plans for NuScale small modular reactors (SMRs) starting operations in late 2020s as well as demonstration project expected to come online in similar timeframes for TerraPower, X-energy, and others, though there is considerable uncertainty about the extent of deployment. Finally, deep decarbonization has the potential to bring greater cross-sector integration and coupling,^16^^,^^17^^,^^18^ including for thermally and electrically integrated hybrid systems with nuclear energy.

Given this context, there are characteristics that are important to reflect in models for all generation technologies, including nuclear energy.

• **Firm capacity:** Firm resources are technologies that contribute to resource adequacy and the planning reserve margin, typically over longer durations.^19^ Low-emitting firm technologies with longer durations include nuclear, carbon-capture-equipped capacity, biomass, geothermal, dispatchable hydropower, long-duration energy storage, and low-carbon gas-fueled plants such as hydrogen turbines or fuel cells. Note that there are varying degrees of firmness and that technologies such as energy storage and variable renewables can have non-zero capacity values depending on system-specific attributes, though these options do not have the same degree of operator control as firm technologies with longer durations. Although nuclear energy competes directly with other “flexible base” generation technologies, recent modeling indicates that there is value for a portfolio of low-emitting firm options,^20^ though their role depends on the availability and costs of substitutes such as long-duration energy storage.^21^ Adequate representations of firm capacity require appropriate temporal resolution^22^ (as discussed in detail in a later section), endogenous capacity contributions,^23^ and operating reserves.^24^
• **Operational flexibility:** Flexibility to quickly change power output, up or down, can be an important operating characteristic. Nuclear technologies with fast ramp rates or that can add thermal storage with molten salts may help the economics of nuclear energy and other resources,^25^ especially with variable renewables increasingly dominating power markets. Load following and ramping are standard features of advanced reactor designs (not fixed output or “must-run” capacity like models have typically assumed) with different levels for existing and new nuclear, where existing reactors can lower output down to 70% of their nameplate capacity within an hour and new nuclear is fully dispatchable.^26^ Despite its load following capabilities, nuclear energy’s economic features (namely its low short-run marginal cost) limit incentives to load follow until deeper decarbonization is pursued^27^ or thermal storage enables higher thermal capacity factors.
• **Product flexibility:** The ability to provide a range of grid services and products can help the economics of individual assets, especially in interconnected energy systems. Many advanced nuclear reactor designs have different coolants and fuels than conventional reactors.^28^ These designs are well-suited for a variety of non-power applications and markets such as desalination, district heating, process heat for industry, or making clean hydrogen or other carbon-free fuels, which may be especially important for analyzing economy-wide deep decarbonization goals.^10^^,^^29^^,^^30^ Using nuclear to provide higher-temperature, zero-emissions heat for industrial processes could be a mitigation approach for costly-to-abate industries, including cement, petrochemicals, refineries, and metal and glass manufacturing.^29^^,^^31^^,^^32^
• **Changes in capital and fixed operations and maintenance (FOM) costs:** These cash flow changes may impact new deployment and retirements of existing capacity. There are questions about capital and operating costs over time and across subsequent installed capacity (e.g., endogenous technical learning), financing, and construction lead times, including how modular design with smaller unit sizes could alter these outcomes.^33^^,^^34^ Modernization for existing nuclear (e.g., automation and improved materials) also may alter these projections.^35^

Some characteristics such as enhanced safety are not directly included in models.^36^ However, potential concerns about safety, waste disposal, recent delays in planning and building plants, project risk, and non-proliferation have contributed to lower recent investments,^31^ and although advanced nuclear designs offer potential enhancements around these issues, many of these factors are challenging to model and create uncertainty about nuclear energy’s future contribution. Although many technologies benefit from direct and indirect government support from policy, financial incentives, and loan guarantees, the unique nature of nuclear energy imposes burdens on the private sector that require public sector de-risking for upfront capital investment in the nuclear fuel cycle and for capping liability in the event of accidents with significant offsite impacts. Given the high capital costs and chronic delays associated with recent nuclear construction projects, government backing of demonstrations and early deployments could be needed to fill gaps in commercial financing.

## Insights from recent deep decarbonization modeling

This section summarizes insights from recent deep decarbonization modeling of the power sector and energy systems, which helps to contextualize factors that matter for modeling nuclear energy.

### Climate policy and technological costs

**Figure 2:**
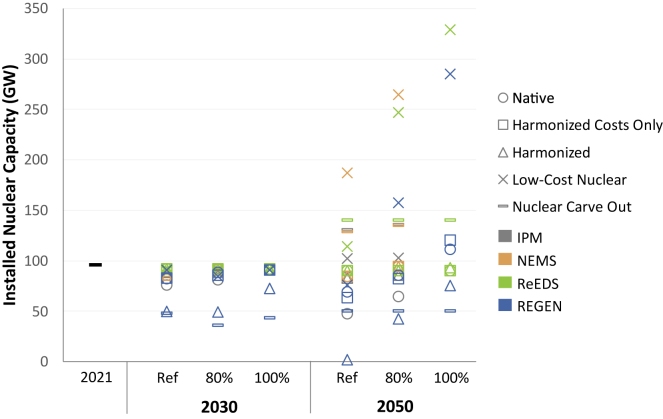
Total installed nuclear capacity in the US by year and policy scenario across all models and technology sensitivities based on Bistline, et al. (2022) Policy scenarios include a current policies reference (“Ref”) and “80%” or “100%” reductions in electric sector CO_2_ by 2050 from 2005 levels. Colors correspond to different models, and shapes correspond to different technology sensitivities. Detailed scenario descriptions and discussions of model results are provided in Bistline, et al. (2022).^37^

Projected nuclear capacity can span a broad range across models and scenario assumptions. Figure 2 shows total installed nuclear capacity over time across a range of models and scenarios based on a recent multi-model comparison.^37^ These experiments use harmonized scenarios to identify the relative roles of input assumptions and model structure on nuclear deployment. The study varies the policy environment across a reference scenario with current federal and state policies in the US, one that adds power sector CO_2_ constraints to reach 80% reductions by 2050 from 2005 levels, and another that reaches 100% power sector decarbonization by 2050. These policy scenarios are conducted under different technological assumptions: 1. Native technology cost and performance assumptions, which models typically assume by default; 2. Harmonized costs only, where all models adopt the same technology costs and fuel prices but use their own assumptions about financing and discounting; 3. Harmonized costs that use the same assumptions about technology costs, fuel prices, financing, and discounting; 4. Harmonized assumptions with low-cost nuclear, where harmonized assumptions for new nuclear are lowered from $5,000/kW by 2050 to $2,000/kW in 2035 and beyond and lower FOM costs for existing nuclear capacity are assumed. Note that these deep decarbonization scenarios assume that a national cap-and-trade policy is used to reach a cost-minimizing mix; however, actual policies and incentives would likely be heterogeneous and impact investments, costs, and emissions.

New nuclear deployment varies considerably based on scenario definitions—the most impactful of which are assumed future nuclear capital cost trajectories and CO_2_ policy assumptions. Decarbonization targets generally help to retain existing nuclear capacity but are typically not enough to bring new nuclear capacity online in the absence of significant cost declines.^37^^,^^38^ Models show sizable nuclear additions in scenarios that layer power sector decarbonization policy with low-cost assumptions for new nuclear capacity (moving from $5,000/kW by 2050 to $2,000/kW in 2035 and beyond). With these low costs, total installed nuclear capacity including existing plants ranges from 76 to 187 GW in 2050 with current policies and incentives (as of late 2021), compared to 285–329 GW under a zero CO_2_ policy.

CO_2_ emissions policy assumptions and policy design, as well as future costs for new nuclear plants, are first-order drivers of nuclear capacity additions and retirements. Details about a policy’s stringency, timing, and technology eligibility influence decarbonization planning and costs. Zero-emissions policies that allow carbon removal technologies (i.e., “net-zero” CO_2_ emissions policies) result in lower deployment of nuclear and renewable technologies relative to policies that do not allow negative emissions options (i.e., “carbon-free” or “absolute-zero” policies). Definitions of eligible technologies, timing of the electric sector zero-emissions target, and nuclear technology costs affect the generation mix.^37^^,^^39^ Even for the same net-zero emissions target, multi-model studies for Asia, Europe, and Latin America suggest that a wide range of nuclear deployment could be possible.^40^^,^^41^^,^^42^ Steep cost reductions can lead to deployment of new nuclear even absent new decarbonization policy. Thus, both the cost and policy assumptions will be central drivers of nuclear capacity additions in long-term models.

Nuclear deployment is also higher in deep decarbonization scenarios where constraints are placed on other technologies such as land use constraints or transmission cost assumptions that increase the costs of available wind and solar resources.^43^ The literature also suggests that nuclear deployment is higher when carbon removal is limited and other clean firm technologies are constrained.^20^^,^^25^^,^^44^ These findings suggest that nuclear energy could provide a hedge against unavailability risks of other low-emitting technologies because of higher-than-expected costs, public acceptance, infrastructure delays, supply chain constraints, or other unexpected technical challenges—assuming nuclear energy is not subject to the same challenges.

Technological characteristics of nuclear energy, beyond its capital costs, also affect its deployment and operations. Several new nuclear designs include thermal energy storage through molten salts,^25^^,^^28^ and such storage systems between the nuclear reactor and generator could allow heat generated by the reactor to be stored and shifted in time, enabling load following capabilities to better match demand profiles. Thermal storage can add to the capital and operating costs of nuclear systems, but the value provided by this storage in displacing natural gas and battery storage (which balance the variability of wind and solar) can lower total system costs by as much as 15% under deep decarbonization policies.^25^ Beyond integrated thermal storage, broader strategies for flexible nuclear plant operations—altering output through ramping and providing grid services such as operating reserves and frequency regulation—can have value by decreasing total power system costs, lowering emissions, increasing revenues for nuclear plants, and reducing wind and solar curtailments.^27^ For existing nuclear, modernization through automation and improved materials can alter operations, maintenance costs, and retirement decisions.^35^

Overall, these results suggest that nuclear energy plays the largest role in scenarios that combine stringent emissions policies, cost reductions, and constraints on the deployment of other technologies, which highlights the model dimensions related to these areas. Note that each of these issues is uncertain, and the probability of occurring in tandem is lower than their individual probabilities. Studies indicate that decarbonization pathways are cheaper when nuclear energy is part of the mix.^12^^,^^20^^,^^45^ Differences in shares may reflect differences in model structure (e.g., temporal resolution, as discussed in the next section), input assumptions (e.g., technology cost and performance), and scenario specifications.

### Deep decarbonization mixes

**Figure 3:**
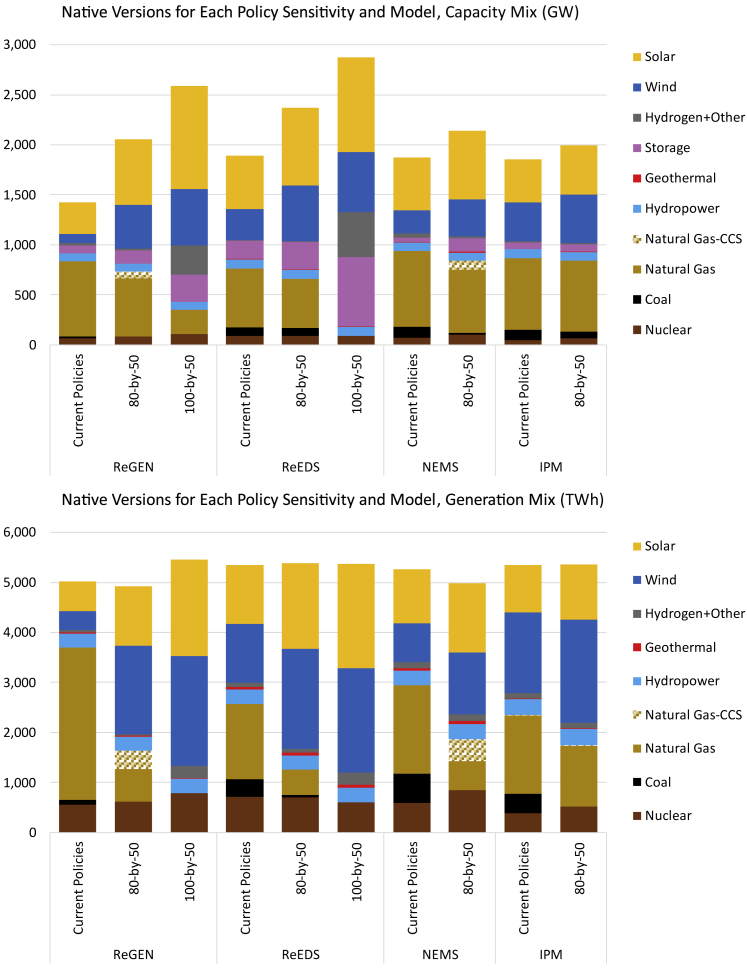
Total installed capacity (top) and generation (bottom) results in 2050 in the US by technology across policy scenarios by model based on Bistline, et al. (2022) Scenarios assume native technological cost and financing assumptions. Detailed scenario descriptions and discussions of model results are provided in Bistline, et al. (2022).^37^

Nuclear provides firm, zero-emissions electricity that could complement large buildouts of wind, solar, energy storage, and other resources that are subject to daily and seasonal variability or energy-limited discharge. With an electric sector policy that reduces CO_2_ emissions by 80% (from 2005 levels) in 2050, there are several consistent findings across models (Figure 3). In particular, these scenarios indicate that the least-cost resource mix typically includes maintaining existing nuclear capacity; lowering coal capacity significantly; and deploying considerably more wind, solar, and energy storage (though magnitudes of these trends vary by model and scenario definitions). These findings generally align with the decarbonization literature.^14^^,^^46^

Many decarbonization studies see nuclear energy and other low-emissions dispatchable technologies as complements to renewable energy technologies. In particular, nuclear’s always-available power can fill multi-day gaps when wind and solar output are low, and the availability of such low-emitting firm technologies can help to lower decarbonization costs.^19^^,^^20^^,^^47^ The declining capacity credit (and value deflation more broadly) of variable renewables and other system resources as a function of their deployment is typically a reason why other technologies come into the mix with deeper decarbonization, even with very high renewable shares. For instance, Cole, et al. (2021) show that, even at 95% renewable generation, roughly half of firm capacity is procured from non-renewable, non-storage resources.^24^

The emphasis on energy security and decreasing dependence on fossil fuels, especially in the wake of Russia’s war in Ukraine, may mean that countries aim to minimize gas-fired generation as they decarbonize.^6^ Recent analysis indicates that this side constraint to eliminate gas in the power sector could lead to greater contributions from nuclear, long-duration energy storage, battery storage, and renewables in the US, though electricity prices increase 12% relative to a decarbonization pathway with gas.^44^

A broad range of generation outcomes is possible, both for nuclear power and other generation options. The range of nuclear deployment (from 2 to 329 GW in 2050 from Figure 2) highlights uncertainties moving forward, but it also stresses the importance of significant nuclear technology advancement and electric sector policies in driving deployment. Ultimately, more diverse portfolios may be valuable in reaching deeper decarbonization targets not only because of the different functional roles of technologies but also considerations related to system costs, reliability and resilience, land requirements, complementary resource needs, cross-sector integration, energy security concerns, and regional differences.

The magnitudes of different electric sector value streams vary by technology, model, region, and scenario. In many instances, nuclear capacity operates with high capacity factors (91–94% annually under a reference scenario and 77–92% under deeper decarbonization) because of its low variable costs, and energy is generally the primary value stream (i.e., bulk electricity sales). However, a stringent CO_2_ policy can shift the capacity value stream (i.e., providing firm capacity contributions during periods of high net demand) and make it larger than the energy value. Regions with supporting policies and lower wind and solar resource quality tend to have higher nuclear deployment, including the Western and Southern US. Ultimately, there is uncertainty for most fuel types and energy technologies, each with its major constraints and policy risks.

Lower nuclear capital costs and lower discount rates lead to higher new builds of nuclear capacity. Assumptions about discount rates and economic lifetimes can materially impact power sector generation and capacity outcomes, especially for nuclear energy given how it is a capital-intensive and long-lived resource. Nuclear and other generation technologies entail intertemporal tradeoffs between upfront capital costs and ongoing operating costs, which are influenced by discounting assumptions. Discount rates have countervailing effects on existing and new nuclear—lower rates increase new nuclear capacity but decrease shares from existing nuclear.

### Impacts of model choices

Model complexity can strongly impact projected electric sector investments and costs, including nuclear energy deployment. Many considerations (e.g., temporal resolution) have more significant impacts with deeper decarbonization. Stringent emissions reduction targets and higher variable renewable energy deployment potentially bring additional resources into the mix, and the economics of many options (including batteries, long-duration energy storage, flexible generation, demand response) depend on the alignment between hourly (or subhourly) net load and output, chronological operations, and interactions across different geographies and portions of the energy system. Specifically, higher temporal resolution is critically important for policy analysis, electric sector planning, and technology valuation in a range of scenarios to capture the joint variability of time-series variables.^22^ Common approaches to simplify temporal resolution—the number of time segments explicitly modeled within a year—in long-term energy models may not reproduce fundamental relationships for power sector decarbonization, including abatement costs rising nonlinearly in decarbonization levels, diminishing marginal returns (especially for variable and energy-limited resources), and value of broader technology portfolios. All else equal, simplified approaches for capturing variability tend to understate nuclear deployment and dispatchable capacity relative to higher-resolution approaches. For instance, a US study investigating strategies for reaching zero emissions in the power sector indicates that a model with hourly temporal resolution includes 117 GW of new nuclear in a cost-minimizing portfolio, whereas models with simplified approaches would not build any new nuclear.^22^ Similarly, a model’s spatial resolution and coverage can capture or exclude transmission-related constraints, which can alter the value of trade, dispatchable generation, energy storage, and demand-side flexibility.^24^

Levelized-cost metrics are incomplete for evaluating the relative competitiveness of system resources.^48^ Comparing traditional levelized-cost metrics between different resources with dissimilar characteristics (e.g., contrasting the levelized cost of electricity for solar and nuclear power) ignores differences in the value of the resources to the system, which become more apparent at higher temporal resolutions. Evaluating the competitiveness of different power system resources and their interactions requires detailed systems models—such as capacity expansion and energy systems models that are the focus of this article—to assess the cost *and* value of the resources under consideration. See^49^ for a more detailed discussion of cost metrics and their appropriateness for different modeling approaches and research contexts.

Model representations of changes in technological performance and costs are critical determinants of model outputs. Technological change can either be exogenous (i.e., based on pre-defined input assumptions about changes over time) or endogenous (i.e., based on model-driven changes in deployment given input assumptions about learning rates). Endogenous technical change can lead to higher or lower projections of new nuclear deployment depending on learning rates for nuclear and other technologies but raises several challenging conceptual and practical considerations,^37^^,^^50^^,^^51^ especially given the path dependence of such processes.

Some model-related factors have smaller impacts on nuclear outputs. For instance, flexibility assumptions about existing and new nuclear technologies have more limited effects on their capacity and generation at least with hourly resolution.^37^ Ultimately, appropriate levels of model detail depend on the type of analysis being performed, motivating questions, available data and resources, system characteristics, and analysis timeframe.

## Modeling Challenges and Needs

**Figure 4:**
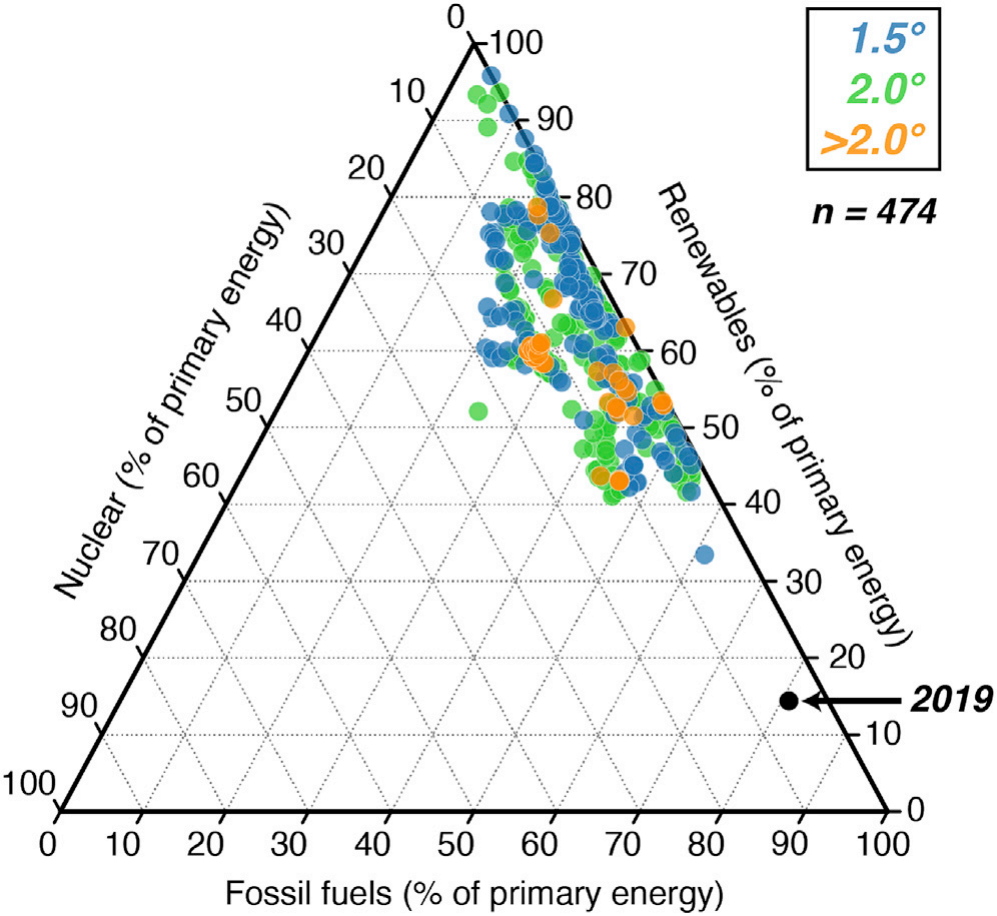
Primary energy resources in global net-zero CO_2_ emissions scenarios Points represent different models and scenarios from the Intergovernmental Panel on Climate Change (IPCC) Working Group III scenario database.^52^ Ternary diagrams show the percentage of primary energy from renewables (right axis), fossil fuels (bottom axis), and nuclear (left axis) for scenarios that limit warming to <1.5°C (blue), 2.0°C (green), and >2.0°C (orange) scenarios. The three axis values for each individual point sum to 100%, and tick marks run parallel to the corresponding gridlines. Primary energy represents the physical energy content of fuels using a direct equivalence approach.

With rapid decarbonization targets under serious discussion by many policymakers, updating models’ representations of nuclear energy and other technologies is increasingly important. Representing characteristics and applications of nuclear energy in systems models can impact assessments of their competitiveness. Nuclear energy has the potential to play an expanded role in net-zero energy systems (Figure 4), though there is some disagreement about the size of this role relative to other technologies. For scenario ensembles like the one shown in Figure 4, models may have limitations that misestimate nuclear shares as fossil fuel consumption drops such as lower temporal resolutions, representing only traditional nuclear, and others discussed in earlier sections. Based on our work and review of the literature, we recommend skepticism for any least-cost decarbonization study that suggests that nuclear is never cost-effective or always cost-effective across a broad range of scenarios.

There are several model development priorities and data needs related to nuclear and broader energy systems analysis to ensure the relevance of models for policy and planning with deeper decarbonization.

• *Providing public data for existing and new nuclear costs:* Given the notable impact that cost assumptions can have on model results (Figure 2), it is important for these costs to reflect the best available information. Public data are lacking for fixed operations and maintenance costs at existing nuclear power plants, and cost assessments of new nuclear technologies are limited and, when provided, only cover a limited number of nuclear technologies—typically a light-water reactor and a small modular reactor. This gap suggests that it would be valuable to have a database of advanced nuclear technological cost and performance assumptions that reflect the diversity of advanced reactor designs. Such a database would have a level of detail similar to the open-source Annual Technology Baseline (ATB) maintained by the US National Renewable Energy Laboratory (NREL). Although cost and performance assumptions are uncertain for other technologies too, the nascent nature of advanced nuclear makes such projections especially uncertain. In addition to input assumptions, it is important to compare model algorithms and heuristics for power plant retirement, cost, and operational decisions against actual data and to update these model features as appropriate. For instance, FOM costs have historically assumed escalation over the age of plants;^37^ however, actual plant data in the US indicates declining FOM and operating costs over the past decade.^53^ FOM costs for nuclear plants can be higher than other technology types, which increases the importance of public data.^35^
• *Sensitivity analysis and uncertainty:* Conducting a wide range of sensitivities to test the robustness of conclusions becomes increasingly important under deeper decarbonization, especially with uncertainties about future policies, technologies, markets, and public acceptance. In light of these uncertainties and the potential hedging role of nuclear, it is also important to understand how the explicit inclusion of uncertainty can alter hedging strategies and can identify robust versus brittle solutions. This may mean explicitly incorporating uncertainty through stochastic or robust optimization and not only conducting sensitivities to alternate inputs, as the hedging role of nuclear and other technologies may be undervalued in a deterministic framework.
• *Representing multi-vector hybrid systems and capturing integration across systems:* Pathways toward achieving a net-zero energy system typically involve growing interactions among electricity supply, energy supply, and energy demand (including electricity, direct fuel use, and heat). Hybrid energy systems—including those that comprise a nuclear power plant and other technologies (e.g., electrolyzer, direct air capture, solar and/or battery technologies) to produce electricity and other energy products—have been proposed as candidate technologies for flexibly contributing to the full spectrum of demands across the energy system.^10^^,^^54^ Such systems can utilize multiple feedstocks and provide multiple products/services, potentially with very low or zero emissions rates. However, the dynamic optimization of these resources is complex owing to their diverse configurations, multiscale interactions, and multiple markets, which makes modeling such resources challenging. Although multi-vector hybrid systems can encompass a range of technologies, advanced nuclear designs can be well-suited for a variety of non-power applications and markets, which makes the consideration of such systems especially relevant to the future economics of nuclear energy.
• *Determining appropriate levels of model complexity and endogeneity for given applications:* There is currently limited guidance about the conditions under which higher fidelity modeling is needed, despite widespread recognition that some model development decisions have first-order impacts on results and require modeler judgment to balance the level of detail with tractability. The literature surveyed here indicates that temporal resolution—not only how many intra-annual periods are modeled but also how they are selected—is a large driver of power sector decisions, especially those related to nuclear energy and other low-emitting firm generation options.^22^ Our modeling experience suggests that it is valuable to create modular structures that allow modelers to test the robustness of their results to alternate resolution decisions. Modelers should test their simplified approaches against a more detailed benchmark (e.g., full hourly model) to determine the conditions under which such simplifications may be appropriate. The long-run emphasis on deep decarbonization in many contexts implies that longer-term analysis (or analysis that reaches emissions reductions of at least 80%) should prioritize temporal resolution at the expense of other model dimensions such as timestep length (e.g., choosing five-year periods rather than two-year periods), time horizon, geographical coverage, and other features. However, modelers may consider prioritizing spatial detail and granularity of existing assets for near-term policy analysis, where the goal is to assess regulatory impacts for specific plants (e.g., performance standards) while capturing system interactions. Another general model challenge and need is to assess suitable levels of model endogeneity, including for areas related to existing and new nuclear—retirements, operations, load shapes, technological change.
• *Representing market incentives faced by electric companies:* Capacity expansion models of power sector investment and dispatch are often formulated in cost-minimization terms across the entire system, subject to policy and technical constraints. However, market incentives faced by asset owners and operators are considerably more complex, fragmented, and heterogeneous than this implicit central planning perspective suggests. In response to these limitations associated with social planner models with perfect information, there is an emerging literature using agent-based models where adaptive decisions by heterogeneous firms may be characterized by imperfect information and bounded rationality.^55^ Models also account for variation in financial characteristics of firms such as the cost of capital and risk tolerance of different electric companies.^56^ Some models also differentiate between cost-of-service and competitive market regions, which can alter firm entry, exit, and operational decisions.^57^ Ultimately, model representations of market incentives require modelers to navigate tradeoffs between descriptive and normative elements (e.g., capacity accreditation based on administrate market rules versus actual contributions), accuracy and parsimony (e.g., because agent-based models require a greater range of parameters and are frequently more computationally intensive), and a variety of modeling and policy objectives (e.g., balancing affordability, environmental goals, reliability, and equity). These considerations are relevant not only for understanding the potential deployment of nuclear energy but also for a broader range of system resources. However, the capital-intensive nature of nuclear energy and importance of appropriately valuing capacity contributions to its deployment make these representations especially relevant for nuclear energy.
• *Modeling alternate country contexts:* Many analyses of nuclear energy’s future role, including the study behind Figure 2, focus on the US. Several unique features stand out about the US and may require new analysis for other geographies, including its relatively low fossil fuel prices, high-quality wind and solar resources, and ample CO_2_ sequestration in many regions, all factors that provide headwinds to new nuclear deployment. In addition, different national circumstances and their associated policy contexts can be influential in encouraging or deterring the deployment of nuclear energy.^58^^,^^59^

### Limitations of the study

With companies, states, and countries targeting rapid and deep decarbonization, many have proposed extending lifetimes of existing nuclear plants and have considered new builds. This work offers a perspective on insights from the decarbonization literature to date, modeling challenges, and analysis needs, which points to several areas for future work.

The literature surveyed in earlier sections indicates that nuclear energy’s future role depends on stringent emissions policies, nuclear cost reductions, capabilities of emerging technologies, regional characteristics, and constraints on the deployment of other resources. Modeling related to these areas becomes increasingly important as net-zero emissions are approached and as greater emphasis is placed on reducing fossil fuel use to ensure the relevance of models for policy and planning. The literature on net-zero emissions energy systems has been growing in recent years but is still relatively new.^60^ Insights about the role of nuclear under these conditions should be updated as a greater variety of country contexts, technological assumptions, and policy scenarios are investigated. In addition, the areas summarized in the “Modeling Challenges and Needs” section point to several modeling gaps related to the representation of nuclear energy and broader energy systems.

Improving models and understanding their differences—including those related to the representation of nuclear energy—can improve planning and policy development to support goals of advancing safe, reliable, affordable, and low-emitting energy.
